# The Size Congruity Effect Vanishes in Grasping: Implications for the Processing of Numerical Information

**DOI:** 10.1038/s41598-018-21003-x

**Published:** 2018-02-09

**Authors:** Gal Namdar, Tzvi Ganel, Daniel Algom

**Affiliations:** 1 Department of Psychology, Ben-Gurion University of the Negev, Beer-Sheva, 8410500 Israel; 2 School of Psychological Sciences, Tel-Aviv University, Tel-Aviv, 6997801 Israel

## Abstract

Judgments of the *physical size* in which a numeral is presented are often affected by the task-irrelevant attribute of its numerical magnitude, the *Size Congruity Effect* (SCE). The SCE is typically interpreted as a marker of the automatic activation of numerical magnitude. However, a growing literature shows that the SCE is not robust, a possible indication that numerical information is not always activated in an automatic fashion. In the present study, we tested the SCE via grasping by way of resolving the automaticity debate. We found results that challenge the robustness of the SCE and, consequently, the validity of the automaticity assumption. The SCE was absent when participants grasped the physically larger object of a pair of 3D wooden numerals. An SCE was still recorded when the participants perceptually indicated the general location of the larger object, but not when they grasped that object. These results highlight the importance of the sensory domain when considering the generality of a perceptual effect.

## Introduction

According to Bertrand Russell’s famous epigram, pure mathematics is the subject that we do not know what we are talking about, or whether what we say is true. The truism granted, mathematics, too, has its pawns, tokens, and moves, and it is the numerals that comprise the abstract, dimension-less tools of the game of mathematics. However, in contrast with their abstract nature in mathematics, in the empirical world every numeral comes dressed in a multitude of physical attributes. Every numeral you have ever seen, heard, or touched came in a certain physical size, shape (font), loudness, or texture. Numerals can even come in the form of graspable three-dimensional objects. Inevitably, a numeral is the union semantic and physical features. If so, it is only appropriate to ask: How do these features interact in cognitive processing? Suppose that you judge the *physical size* of a numeral presented for view. Does the numerical magnitude of the numeral influence your perception of size? Such an influence is consistent with the popular idea that numerical information is activated in an automatic fashion just whenever a numeral is presented for view for any purpose. However, the effect of irrelevant numerical magnitude is often absent in judgments of size, thereby challenging the alleged automaticity of numerical perception. A novel attempt at resolving the nature of numerical processing forms the overriding theme of the present study.

The issue is not new, of course, but here we address it by combining ideas and methods from two separate domains. The first domain is that of visual perception, in particular visual selective attention: Can people focus exclusively on physical size while ignoring numerical magnitude? If they can, they then select the *physically* larger member of the pair 7 2 (congruent stimulus) as fast as that in the pair 7 2 (incongruent stimulus). If they cannot, performance is better with the first than with the second pair, yielding the typical measure of the size congruity effect (SCE). Because the SCE frequently obtains, it is considered by many to be a marker of the automatic activation of numerical information. The problem is that the SCE is absent in a fair number of studies. Moreover, its absence is not haphazard or random, but rather the orderly outcome of the experimental design used. Slight variation of the stimuli and the design are capable of producing an inflated SCE, of eliminating the SCE altogether, or of reversing it such that physical size now intrudes on numerical perception. The upshot is, the question of automatic activation of numerical magnitude is not fully settled within visual perception and attention. It is at this juncture that evidence from a second domain, grasping, becomes valuable.

It is increasingly recognized that our sense of vision subserves two separate functions: perception and action. Vision is used in the first sense when you assess (subjectively) the size of a chair in the shop to decide if it fits your room. Vision is used in the second sense when you reach to the chair with your hands (e.g., to lift it after purchase). To underscore the difference, the function of vision in the second instance is to guide motor *action* by the viewer, rather than to enable mere perception or a perceptual report. Now, all SCE studies to date are based on visual *perception*, not visually guided *action* – the pertinent studies did not include a motor component by which the participant extends her hands to hold the stimulus. The present study fills this lacuna by subjecting the hypothesis of automaticity to testing by grasping. This test is singularly severe because grasping has been shown to be immune to effects of task-irrelevant features or factors of context. For example, grasping has been shown to be exempt from such compelling visual (perceptual!) illusions as those of height-width illusion or the Ponzo. If the SCE survives responding via grasping, the hypothesis that numerical information is activated in a mandatory fashion is lent powerful support.

In the remainder of the introduction, we first establish the perception-action partition of visual functions. We then summarize relevant research from vision-for-perception and from vision-for-action by way of motivating the present study. We believe that our approach and findings is bound to revitalize the debate on the key cognitive question of how people process semantic information.

### The Perception-Action Complementarity

The distinction between the two visual systems is supported by a plausible anatomical substrate and systematic differences in operation characteristics. According to an influential idea suggested by Goodale and Milner^1^ the visual system is segregated, anatomically and functionally, into two visual pathways. In this view, the ventral pathway supports visual *perception*, while the dorsal pathway subserves goal-directed *action*. The evidence supporting this model is strong, based on multiple instances of double-dissociation^2–8^ (but see^9,10^). Corresponding to the anatomical distinction are characteristic differences in modus operandi. The perceptual system is notorious for its susceptibility to contextual information. For example, people are unable to ignore the meaning of a (color) word when naming its print color (the Stroop effect, see^11,12^ for reviews), are unable to ignore the object’s height when judging its width (Garner interference^13^, see^14^ for review), and are unable to ignore the object’s size when estimating its weight (the size-weight illusion^15^). Familiar visual-perceptual illusions (e.g., the Ponzo or Ebbinghaus) are further instances of contextual influence on perception. The SCE, when it is present, provides another example of the difficulty at ignoring task-irrelevant information in perception, this one in the numerical domain. It takes people longer to select the physically larger member of a pair of numerals when this member is numerically smaller than when it is also numerically larger.

In contrast with mere perception, actions guided by vision seem largely exempt from contextual influences. It is repeatedly found that reach-to-grasp kinematics are unaffected by context because the dorsal stream visual network acts via exclusive focusing on the to-be-held object, preserving its absolute size (e.g.,^16^ and references therein). It is due to this effector-specific absolute mode of operation that grasping is largely resistant to visual illusions. In fact, mere intention to grasp suffices to activate the action system. By contrast, viewing passively the same stimuli fools the perceptual system because the ventral system relies on relative metrics within scene-based reference frames.

Concerning visual illusions in particular, Vishton *et al*.^17^ showed that the magnitude of the Ebbinghaus illusion decreases when the observer is asked to grasp or reach for the target rather than merely report its size. If the observer is asked to throw balls at the target, the perception of the target’s size is correlated with the number of the successful hits^18^. Visual search of features such as object orientation has been shown to require less saccades when participants *intend* to act upon the searched items^19^. In this study, we tested the effect on performance of both action and of the intention to act.

### Vision-for-Perception: Is Numerical Magnitude Processed in a Mandatory Fashion?

The SCE is regularly observed in studies of numerical perception (e.g.,^20–27^). Given its recurrence, the effect is often taken to reflect on the mandatory processing of numerical magnitude (e.g.,^28–30^. However, systematic biases in the standard experimental design call into question the robustness of the SCE, and consequently, the obligatory nature of numerical processing. Little stimulus alchemy suffices to eliminate the SCE or reverse the effect such that physical size intrudes on perception of numerical magnitude more than vice versa (the reverse-SCE^23^). Algom *et al*.^20^ (see also^14^) have identified two critical biases prevalent in published SCE studies. First, there is a glaring asymmetry in the number of stimuli used for the numerical and the physical dimensions. Typically, the numbers 1 to 9 (inclusive) are used for the former, but only two or three values (small, medium, large) are used for the latter. As a result, virtually all pertinent research pitted a finely grained numerical dimension against a coarse physical dimension. This asymmetry itself can determine the observed interaction (=SCE). Melara and Mounts^31^ (see also^12,32^) have shown that the mere number of stimuli on an irrelevant dimension affects classification performance on the relevant dimension.

For another bias, the relative discriminability of values along the number and the size dimensions was not matched. Matched discriminability or salience means that the time and accuracy needed to tell apart values along the number dimension is the same as those needed to tell apart values along the size dimension. However, mismatched discriminability favoring numbers was present in virtually all studies of the SCE. Values along the number dimension were much more discriminable from one another than values along the size dimension (e.g., it took participants much longer to tell the small and large physical sizes apart than to tell the numbers 4 and 5 apart). The presence of this asymmetry is critical because the more discriminable dimension will disrupt performance on the less discriminable dimension more than vice versa^31,32^. In the present case, irrelevant numerical values affected performance with physical size (=SCE) not because they are activated in an automatic fashion but simply because the values of number differed perceptually from one another more than did the values of physical size from one another. Notably, when care was taken to match discriminability (and number of values along the two dimensions), the SCE collapsed. And, when physical size was purposely made more salient than numerical value, a reverse SCE emerged^12,20,23,24,26,27,32^. The malleability of the SCE casts doubt on the automatic nature of processing numerical magnitude. Recently, Sobel and his coworkers^33^ have found that number and size are processed in an independent fashion, a result inconsistent with automaticity. Are there further means to resolve the issue of mandatory processing of numerical magnitude?

### Perception-for-Action: Context Independence when Grasping Numbers?

Grasping a part or some property of a multidimensional object has been found free of interference from task-irrelevant features of the same stimulus, although such interference is present in visual perception^3,13^. Major visual-perceptual illusions diminish or disappear altogether when the responses are motor actions under direct visuomotor control^7,34–40^ (but see^10,41,42^). Grasping is immune to contextual information to the extent that it has been shown to violate even such a fundamental psychophysical principle as Weber’s Law^36,43–45^. If context independence prevails, then the SCE should vanish when people grasp numbers of different physical size, i.e., numerical magnitude should not affect the grasping response. Here we asked: Does the alleged automaticity in perceiving numerical information survive the change in response mode? If numerical magnitude is always processed, then the SCE should also emerge in grasping.

## The Present Study

The main goal was to subject the SCE to a stringent test by a modality known to be immune to task-irrelevant information. Observing the vanishing of the SCE in grasping would add to the growing doubts about its obligatory nature^46^. Simultaneously, this observation would further support the context independent nature of the action system. This much granted, a stringent test invites equally stringent controls to exert its full impact. The control condition is required by the nuanced relation between grasping and motor responding. Note that grasping always involves motor responding mediated by the dorsal action system, but that the reverse is not true. All motor responses are not also those of grasping and are not mediated by the dorsal action system. Suppose that you are presented with two bottles and asked to select the larger one. When you do so by grasping the larger bottle, it is likely that your object-directed response is mediated by the dorsal action system. When you do so by pointing to the direction of the larger bottle with your finger (without holding it and without any plan of holding it), it is likely that your response is mediated by the ventral perceptual system. In point of fact, the latter example is on a par with oral or keypress responding conveying the person’s perception. Therefore, it is important to dissociate the influence of actual grasping from mere motor reaction without grasping or any intention of grasping the object. Again, this latter condition is actually that of perception, the motor movement notwithstanding.

In Experiment 1, we pioneered the crucial examination of the SCE by grasping. The participants grasped literally the larger real-life object depicting a numeral. In the perceptual control condition, the participants made the same decision (i.e., selecting the physically larger object), but they conveyed their response by tapping on the thigh in the side of the selected object. This perceptual control condition was also included to test for the possibility that people simply ignore the numerical information embedded in the objects.

In Experiment 2, we used a modified perceptual size-congruency paradigm aimed at reducing reaction times in order to equate RTs with the grasping condition in Experiment 1. This was done in order to test whether the pattern of results obtained in Experiment 1 in the perceptual task could have been attributed to potential differences in reaction times between the grasping and the perceptual task. In Experiment 3, we expanded the arsenal of measures to include on-line kinematic recording of the full trajectory of movements in the two conditions. To anticipate the main finding, the results of all three experiments converged on the conclusion that the SCE is evident in perceptual estimations but vanishes in grasping.

## Experiment 1: Grasping Congruent and Incongruent Objects

### Method

#### Participants

A group of 24 right-handed students from Ben-Gurion University of the Negev, who gave their informed consent, participated in the experiment (11 males; mean age = 25.04, SD = 2.21). The experimental protocol was approved by the ethics committee of the Department of Psychology in Ben-Gurion University of the Negev. The study adhered to the ethical standards of the Declaration of Helsinki.

All participants signed a consent form prior to their participation in the experiment. The manuscript contains no information or images that could lead to identification of a study participant.

#### Stimuli and Design

The stimuli consisted of wooden objects cut in the form of the Arabic digits 2 and 8. Each digit-form was produced in two values of size, large (50 × 32 mm in height and width) and small (30 × 18 mm). The third dimension was constant at 5 mm. Factorial combination of number and size created the current set of 4 stimuli: a physically large object in the shape of a numerically large number, a physically large object in the shape of a numerically small number, a physically small object in the shape of a numerically large number, and a physically small object in the shape of a numerically small number. Note the first and last of these objects comprise congruent stimuli, whereas the two in-between comprise incongruent stimuli.

**Figure 1:**
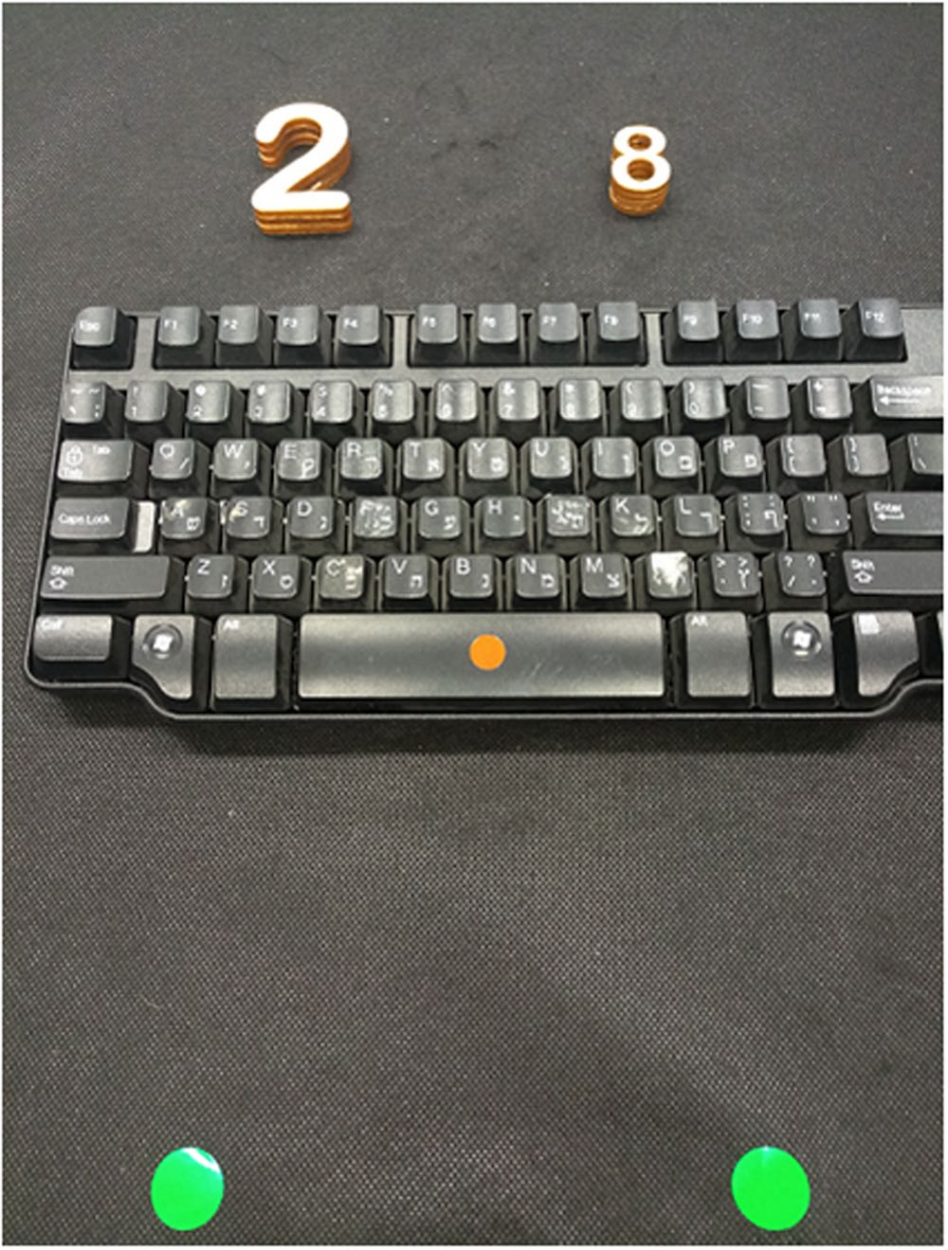
An example of a (incongruent) trial. The participant indicated which of the two digit-shaped objects was physically larger. Starting position is shown by an orange sticker on the spacebar key. The pair of green stickers at the bottom was used in Experiment 3 to indicate general lateral location.

On each trial, a pair of objects was placed in front of the participant and she indicated which of the two was *physically* larger (see Fig. 1). The experiment included two conditions. In the object-directed movement condition (ODM), the participant reached for the physically larger object with the right hand, grasped and lifted it using the index finger and the thumb. In the non-directed movement condition (NDM), the participant used her index finger and thumb to touch either the right or left thigh, corresponding to the location of the physically larger digit. Note that the NDM condition entailed neither grasping nor intension of grasping. Each condition consisted of a block of 96 trials (half congruent, the other half incongruent), preceded by 10 practice trials. The order of conditions and order stimulus presentation within a condition was random and different for each participant.

#### Procedure

The participant was sitting in front of a black table top with the tips of the index finger and thumb of the right hand closed together and pressing on the Spacebar key as the starting point. The participant wore a set of LCD goggles (Translucent Technologies, Toronto, ON), with liquid-crystal shutter glasses that were used to control for stimulus exposure time. The experimenter was present in the room and placed the stimuli on the top of the table to start each trial. The stimulus objects became visible (i.e., the trial started) only with the opening of the goggles. Two stimulus objects were placed side by side, 120 mm apart. The midpoint between the stimuli was located at a distance of 150 mm from the starting point (see Fig. 1 again). The tasks were speeded and the participants were asked to respond as speedily and accurately as possible.

#### Data Analysis

The dependent measure was reaction time. We measured the RT of releasing the spacebar upon presentation of the stimulus objects (i.e., from the opening of the glasses). For each participant under each condition, trials with reaction times 2.5 SD below or above average were removed from the analysis (a total of 3% of the trials). RTs were collected using MATLAB (Mathworks, Natick, MA) and Psychtoolbox (http://www.psychtoolbox.org/). Accuracy levels in both tasks were near 100%.

### Results

**Figure 2:**
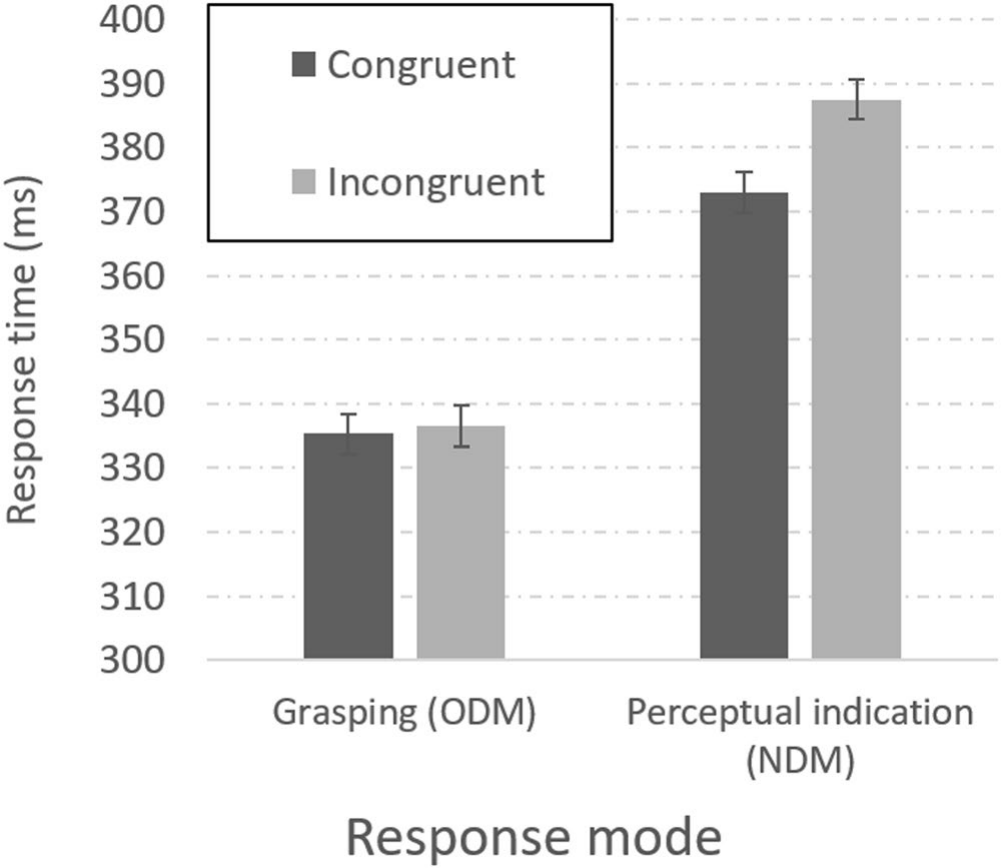
Experiment 1: Reaction times to initiate movement towards congruent (dark bars) and incongruent (bright bars) stimuli for grasping (ODM) and for perceptual indication (NDM). Error bars represent 95% confidence intervals.

Figure 2 gives the results. The crucial data are presented at the left-hand half of Fig. 2. When the observer grasped the physically larger object, the number imparted by its shape did not make a difference. Incongruent and congruent objects were separated by a negligible less than 2 ms difference with average response times of 336.5 and 335.2 ms, respectively (F < 1). Clearly, the SCE vanished in grasping. A Bayesian analysis further supported the parity with a Bayes factor (BF_01_) of 2.77, indicating that the null hypothesis was 2.77 times more likely than the alternative. Our observers noticed the number under the current presentation as is revealed by the results in the condition in which the observer merely indicated the lateral location of the physically larger object (a perceptual response, right-hand half of Fig. 2). In the NDM condition, the responses took longer for incongruent than for congruent objects [388 and 373 ms, respectively, F(1,23) = 19.27, p < 0.001, η_p_^2^ = 0.45, BF_10_ = 94]. The SCE thus emerged in this condition. The interaction of task (grasping, directing) and object congruity (congruent, incongruent) further supported the vanishing of the SCE in grasping [F(1,23) = 18.7, p < 0.001, η_p_^2^ = 0.45]. A main effect was also found for task [F(1,23) = 20.3, p < 0.001, η_p_^2^ = 0.47, BF_10_ > 1000] with RTs for grasping faster by 44 ms, on average, than those in the perceptual indication task. We discuss the source and implications of this difference between the two tasks in Experiment 2 and in the results section of Experiment 3 in which a similar difference is observed.

### Discussion

The evaporation of the SCE in grasping is the signature of the present results. The implications are far-reaching. At minimum, the results indicate that the SCE may be confined to visual perception; it is certainly missing when the response entails direct visuomotor control. It would be easy to dismiss the significance of these results as another example of the insensitivity of grasping to competing task-irrelevant information. On this view, it can be argued that the observers did not even notice the number information. However appealing, this reasoning is not valid. The data in our non-grasping, perceptual condition (done with the same stimuli under the same viewing conditions) show that the same participants were eminently aware of the numbers embedded in the objects (see^47,48^ for similar results). We conclude that the breakdown of the SCE in this experiment is a genuine result.

Is the SCE, hence the activation of numerical magnitude, mandatory under all contexts? Our results show that it is not. Our data thus join several demonstrations from visual perception itself in challenging the automaticity assumption in numerical perception. Automatic processing of numerical magnitude is a contingent phenomenon, not a necessary one.

One obvious reservation with respect to the conclusions drawn is the difference in absolute RTs between perception and action. Those for perception were longer than those for grasping. Consequently, the absence of the SCE in grasping can possibly be attributed to this faster responding, rather than to the different modality employed. Experiment 2 was planned to test for this possibility.

## Experiment 2: Fast Perceptual Estimation of Congruent and Incongruent Images

Our goal in this experiment was to test for the presence of the SCE in perception when responding is fast as that observed in grasping in Experiment 1. To generate very fast perceptual responses, we introduced three modifications of the task used in Experiment 1. First, we used a computerized version of the SCE task with the images of the same 3D objects appearing on the computer screen. Second, the participants responded to the larger physical image by pressing the appropriate key. Note that these features comprise the standard way of assaying the SCE in visual perception (when there is no interest in another modality). Third, we motivated our participants for fast responding by intruding a time-window at the end of which a disagreeable sound was presented to signal “sluggish” reaction. We asked: Would an SCE still observed under very fast perceptual responding?

### Method

#### Participants

An independent group of 24 right-handed students from Ben-Gurion University of the Negev, who gave their informed consent, performed in the experiment (6 males; mean age = 23.1, SD = 1.22). The experimental protocol was approved by the ethics committee of the Department of Psychology in Ben-Gurion University of the Negev. The study adhered to the ethical standards of the Declaration of Helsinki.

#### Stimuli and Design

The stimuli consisted of 2D images of the Arabic digits 2 and 8. Each digit-form was produced in two values of size, large (50 × 32 mm in height and width) and small (30 × 18 mm). Factorial combination of number and size created the current set of 4 stimuli: a physically large object in the shape of a numerically large number, a physically large object in the shape of a numerically small number, a physically small object in the shape of a numerically large number, and a physically small object in the shape of a numerically small number. Note that the first and last of these objects comprise congruent stimuli, whereas the two in-between comprise incongruent stimuli.

On each trial, a pair of images of objects was presented to the participant and she indicated which of the two was *physically* larger by pressing one of two corresponding keys on a keyboard. The experiment consisted of one block of 96 trials (half congruent, the other half incongruent), preceded by 10 practice trials.

#### Procedure

The participants were sitting at a distance of 60 cm from a 21″ computer screen, with their right and left fingers resting on the “k” and “s” keys of the keyboard. In each trial, the two objects were presented side by side, 120 mm apart. The participant wore headphones in which a buzzing sound was played in the end of each “slow” trial in which the participant failed to respond faster than 350 ms following stimuli presentation. The purpose of the buzzing sound was to motivate the participants to produce swift responses.

#### Data Analysis

The dependent measure was reaction time. We measured the RT of pressing the correct key upon presentation of the stimuli. For each participant, incorrect trials or trials with reaction times 2.5 SD below or above average were removed from the analysis (a total of 7% of the trials).

### Results

The mean RT in the present perceptual-judgments experiment was 337 ms. In the face of this very speedy responding, we still witnessed a reliable SCE of 12 ms [means of 331 (SE = 5.7) and 343 (SE = 6.3) ms for congruent and incongruent images, respectively; t(23) = 5.86, p < 0.0001, BF_10_ > 1000]. Note that the present overall mean of 337 ms in perception is comparable to the overall mean of 336 ms in grasping observed in Experiment 1(t < 1, BF_01_ = 4.67). Despite the parity in the absolute RTs, an SCE was still evident in the perceptual judgments of this experiment. Therefore, the longer average RTs in the perceptual task of Experiment 1 cannot account for the different patterns observed with respect to the SCE across perception and action. Accuracy of responding in Experiment 2 was 93% for congruent images and 90% for incongruent images, which rules out an explanation for RT by a speed-accuracy tradeoff.

RTs in Experiment 2 were 337 ms on average, compared to 380 ms on average in the perceptual condition of Experiment 1. The validation of the manipulation was further supported by a significant independent groups t-test between the average RTs of the perceptual conditions of Experiment 1 and Experiment 2 [t(46) = 3.33, p < 0.01, BF_10_ = 20.31]. Importantly, no significant differences were observed between the average RTs in the grasping condition of Experiment 1 (336 ms) and (the perceptual condition in) Experiment 2 [t(46) < 1, NS, BF_01_ = 3.46].

### Discussion

Our modifications of the perception task proved effective in reducing the RTs to the level of those observed for grasping. With these matched RTs, we still observed an SCE in perception, whereas none was observed in grasping (Experiment 1). Consequently, overall faster responding cannot account for the absence of SCE in grasping.

Given the theoretical weight of the results of Experiments 1–2, replication and extension were certainly invited. According to one reservation, the participants in the grasping condition in Experiment 1 initiated their movements before deciding which object was the target, thereby bypassing the SCE. When touching the thigh in the perceptual task, the same participants decided the target object prior to movement initiation, thus displaying the SCE. Therefore, in Experiment 3, the participants were asked in the perceptual condition (NDM) to indicate their choice by reaching to a location at the side corresponding to the target (rather than tapping their thigh). Too, we used a single dependent variable in Experiment 1, release RT. It is possible that other measures show a different pattern (consistent with the SCE). To expand our range, in Experiment 3 we used tasks that were similar to those of Experiment 1, but with finger and hand motion tracked. We thus recorded kinematic trajectory data for the entire movement in order to detect potential emergence of the SCE throughout movement execution. Apart from the kinematic recording, we also measured the RT to releasing the spacebar. A final extension entailed type of judgment. In Experiment 3, we tested for both “larger” and “smaller” judgments (not merely “larger” judgments).

## Experiment 3

### Method

#### Participants

An independent group of 36 right-handed students from Ben-Gurion University of the Negev, who gave their informed consent, performed in the experiment (7 males; mean age = 22.86, SD = 1.24). The sample size was determined using G*power^49^ based on the effect sizes measured in Experiment 1. The experimental protocol was approved by the ethics committee of the Department of Psychology in Ben-Gurion University of the Negev. The study adhered to the ethical standards of the Declaration of Helsinki.

#### Stimuli and Design

The same stimuli from Experiment 1 were used. However, Experiment 3 entailed a between-subjects design with a random half of the participants selecting the physically larger object, whereas the other half selecting the physically smaller object. Within each group, the participants performed once in the grasping (ODM) and once in the perceptual (NDM) condition. In the latter condition, the participants touched one of the colored stickers located beneath the starting position, 75 mm to the left and to the right.

#### Kinematic Recordings

An Optotrak Certus device (Northern Digital, Waterloo, ON) was used to record hand and fingers trajectories. One infrared light-emitting diode was placed on the wrist of the participant’s right hand, and an additional two were placed on the right-hand thumb and index finger (located on top of the center of the distal phalanges). Data regarding the location of each diode was collected throughout the entire movement trajectory at a 200 Hz sampling rate. Movement onset was determined by the release of the starting spacebar. Movement offset was determined as the point in time in which the wrist diode’s speed fell below 50 cm per second for more than 50 ms. Trials were excluded from further analysis if the infrared diodes placed on the participant’s fingers were not visible to the camera while moving towards the target. This resulted in approximately 3% of the trials being excluded. Data from the thumb and index finger markers were collected to assist us in situations in which the movement data could not be used to conclusively determine movement offset. No such trials were identified in the current study, so data from both markers were not further analyzed and only data from the wrist diode was analyzed.

#### Trajectory Analysis

Movement trajectory analysis was performed by examining the lateralization of the movement, i.e., the trajectory on the X axis (from the participants’ point of view). For both grasping (ODM) and perception (NDM) conditions, each trial was normalized in time and then divided into ten segments of equal length, resulting in 11 time points (0–100% of movement trajectory). The effect of congruity on trajectory lateralization was calculated as the spatial difference between congruent and incongruent trials for both target locations (left-right). Higher lateralization values reflect a larger trajectory difference in the direction of the location of the incorrect target (hence, a momentary SCE). Movement times were also calculated as the time interval between movement onset and offset under both experimental conditions.

#### Procedure

The procedure was that used in Experiment 1 with for two notable exceptions. First, each participant was assigned to either the ‘chose larger’ or the ‘chose smaller’ group. Second, in the non-grasping condition, the participants touched a sticker near the hands (rather than their thigh as in Experiment 1). In each group, each participant performed in the grasping and the perceptual conditions. Order of condition and order of presentation within condition was random. This experiment included continuous recording of movement in both groups and conditions. Nevertheless, the tasks were speeded with the participants asked to respond as speedily and accurately as possible.

### Results

**Figure 3:**
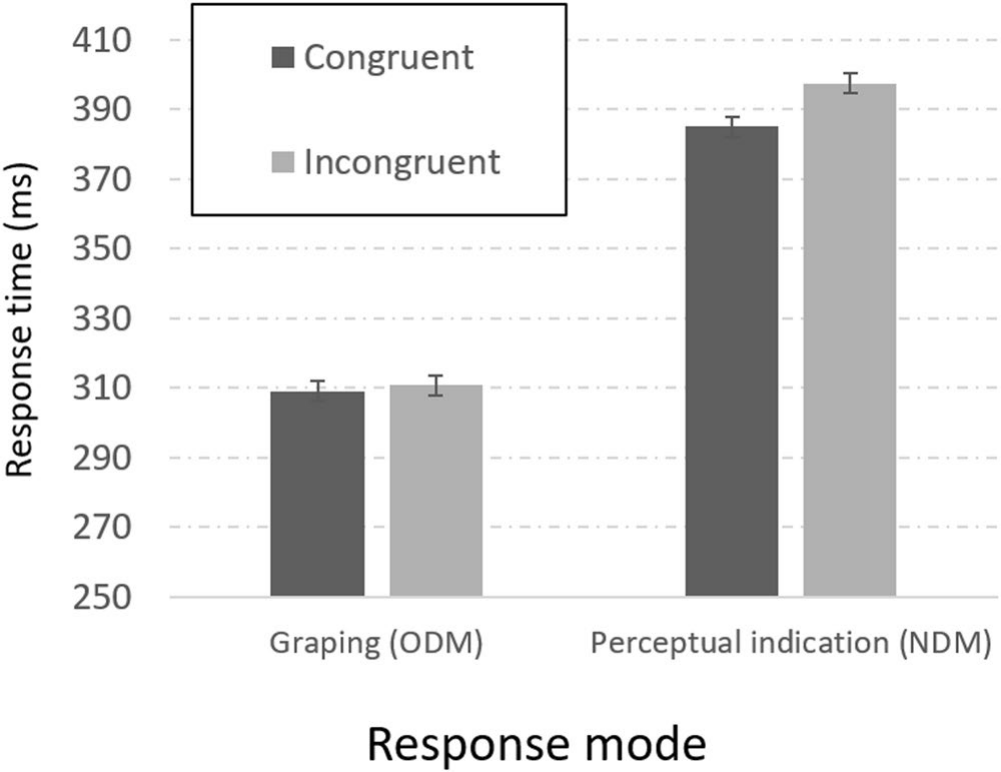
Experiment 3: Reaction times to initiate movement towards congruent (dark bars) and incongruent (bright bars) stimuli for grasping (ODM) and for perceptual indication (NDM). Error bars represent 95% confidence intervals.

*Reaction Times–* Fig. 3 provides the data, Although ‘choose larger’ responses were faster than ‘choose smaller’ responses [F(1,34) = 7.48, p < 0.01, η_p_^2^ = 0.18], this variable did not interact with any of the other main factors. Consequently, we collapsed the data across instructions (groups), so that Fig. 3 shows the combined results over the two groups. The most significant result appears again at the left-hand half of Fig. 3: The mean RTs did not differ between congruent and incongruent stimuli when the participants were required to grasp the object [309 and 311 ms, respectively. F(1,34) = 0.86, NS, BF_01_ = 3.03]. The absence of the SCE in grasping replicates the findings of Experiment 1. Our participants did take note of the numbers that shaped the objects, as evinced by the presence of the SCE in the perceptual condition [385 and 397 ms for the congruent and incongruent trials, respectively. F(1,34) = 11.6, p < 0.01, η_p_^2^ = 0.25, BF_10_ = 18.9], but this did not affect their speed of grasping. The breakdown of the SCE in grasping does not derive from a failure to notice the numerical information. This information is perceived, but is not activated to block (or to assist) the grasping response aimed at object size. The absence of the SCE in grasping (but its presence in perception) is further supported by a significant interaction of Task and Congruity [F(1,34) = 14.55, p < 0.001, η_p_^2^ = 0.30]. A large difference between tasks (81 ms) favoring grasping was again observed [F(1,34) = 106, p < 0.001, η_p_^2^ = 0.75, BF_10_ > 1000].

**Figure 4:**
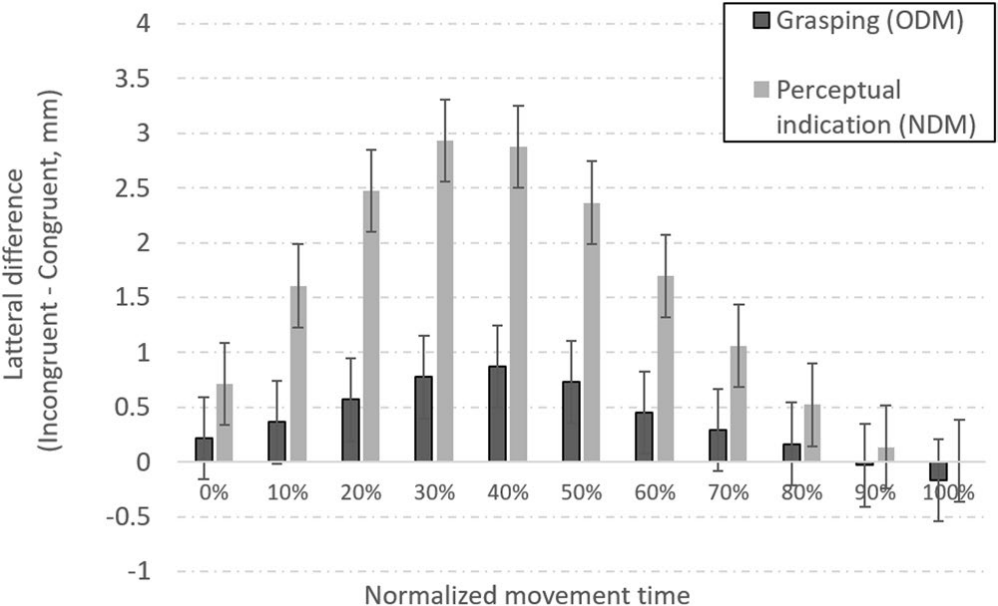
Mean difference in momentary lateral drifts toward the incorrect target between congruent and incongruent stimulus objects. Error bars represent 95% confidence intervals.

*Movement Trajectory*– The dependent variable in this analysis was the difference in direction of the movement between the target and the non-target objects. This difference in momentary lateralization was measured in each of the 11 segments of the movement. Again, instructions to choose the larger or smaller object did not make a difference [F(1,34) = 1.72, p = 0.20, η_p_^2^ = 0.05] nor did they interact with any of the other variables. Concerning the SCE, movement drifts toward the incorrect target were larger for incongruent than for congruent objects in the perceptual condition [1.49 mm, on average, [F(1,34) = 10.64, p < 0.01, BF_10_ = 3.23], but such drifts were not statistically reliable in the grasping condition [0.38 mm, on average, F(1,34) = 2.61, p = 0.12], BF_01_ = 11.47, despite a very small drift during the middle part of the movement. The main effect of movement trajectory [F(10,340) = 131.45, p < 0.001, η_p_^2^ = 0.80, BF_10_ > 1000] documented the fluctuation in the momentary SCE over the course of the movement (see Fig. 4), the SCE being largest in the middle range. Nevertheless, the interaction between task (grasping, perception) and movement segment [F(10,340) = 25.83, p < 0.001, η_p_^2^ = 0.43] pointed to some residual differences in the evolution over time of the SCE in grasping (ODM) and perception (NDM).

*Movement Time*– The overall duration of the movement until reaching the object or the sticker was the dependent variable in this analysis. Once again, instructions to choose the larger or the smaller object did not affect movement time nor interacted with the other factors. There was an overall effect of congruity (=SCE) of a rather small magnitude [7.5 ms, F(1,35) = 18.6, p < 0.001, η_p_^2^ = 0.34, BF_10_ = 0.2], which was only marginally qualified by an interaction with task [F(1,35) = 2.58, p = 0.11, η_p_^2^ = 0.07]. We thus recorded a tenuous SCE of 5 ms in grasping [F(1,35) = 6.3, p = 0.016, BF_10_ = 2.89], half its value in the perception condition [10 ms; F(1,35) = 16.34, p < 0.001, BF_10_ = 81.75]. In conclusion, minuscule SCEs in grasping were only detectable with overall duration of movement.

*The RT difference between tasks*– As in Experiment 1, grasping was faster than pointing. We note though that in Experiment 2 we managed to match the RTs between the grasping task and the perceptual task and still observed an SCE in perception. We also note that the pattern of faster RTs in grasping compared with perceptual estimation has been documented in the action literature^13,50^. Nevertheless, to further support the hypothesis that the difference in the appearance of the SCE does not derive from the difference in the absolute RTs, we performed the following statistical analysis on the data of experiments 1 and 3. The RT data in each task was divided into two groups separated by the median, thereby creating slow and fast distributions of responses. We next considered the slow *grasping* trials with a mean of 354 ms (compared with a mean of 284 ms for the fast grasping trials) and the fast *perception* trials with a mean of 347 ms (compared with a mean of 427 for the slow perception trials), so that no longer was there a difference in speed of response initiation. The data from the two distributions were subjected to an ANOVA with Task and Congruity as within-subject factors and experimental task as a between-subjects factor (3 levels). The results confirmed that the grasping and perception were now comparable in speed (F < 1, BF_01_ = 3.12) yet we still detected the critical interaction between Task and Congruity [F(1,57) = 5.2, p = 0.026, η_p_^2^ = 0.08]. These results confirm that the SCE was present in the perceptual condition but not in the grasping condition, even when the reaction times of the two tasks are similar.

### Discussion

The results of Experiment 3 replicate and extend those of experiments 1–2, using a larger arsenal of measures. The RTs recorded for starting motion fully replicate the pattern observed in Experiment 1: The SCE was present when grasping was not involved, but it was conspicuously missing when grasping the object was the response. The motion trajectory analysis supported this difference, in particular that of the absence of the SCE from grasping, in a powerful way. Think of a trial in which a physically large 2 is presented on the right and a physically small 8 on the left. The participant was expected to move her hand along a (relatively) straight line from the starting position to the sticker located to her right side. The relatively large drift values recorded under this non-grasping condition mean that the participant did not move her hand along such a trajectory, but that the trajectory was drifted to the left side toward the location of the non-target (numerically larger) object (=momentary SCE). Such drifts were statistically absent in grasping. Nevertheless, the trajectory analysis shows that in neither condition did the participants fully decide on the target in advance of movement and that, in each case, the decision was fine-tuned in the course of motion.

The single measure evincing an effect of task-irrelevant numerical magnitude (=SCE) was that of overall motion time. The longer duration recorded with the incongruent displays reflects the extra time consumed by the drift toward the incorrect target. Such drifts were less frequent and smaller when the subsequent task entailed grasping compared with visual perception (see Fig. 4 again), but they did nonetheless produce a small SCE in overall time in grasping, too. The trajectory analysis does not pinpoint any particular epoch that qualitatively distinguishes grasping and perception motion. Consequently, we conclude that the motion time data are in general agreement with the RT data in showing a small or a missing SCE under grasping throughout all aspects of responding.

## General Discussion

Numerical information is believed to be processed in an automatic, unavoidable fashion. According to Dehaene^28^, “The presentation of an Arabic numeral elicits an automatic activation of the appropriate… magnitude code… [that] cannot be repressed, even though magnitude information is irrelevant to the task (p. 21). Or, in Carr’s rendition^51^, “Meaning is activated automatically… just about whenever a word [number] is processed for any purpose” (p. 216). It is on this background that the SCE has been considered a classic marker of the obligatory nature of numerical information processing: Deciding the physically larger font of a pair of numerals is affected by the numerical value of the numeral forming the font. However, a growing counter-literature challenges the automatic hypothesis, demonstrating that the SCE is avoidable, even reversible (with physical size intruding on judgments of numerical magnitude more than vice versa). It is at this juncture that information from another domain, visuomotor control and grasping, is welcome.

The SCE emerged in the current study when the participants indicated the physically larger number-object in perceptual tasks that entailed tapping on their thigh, pressing a key, or touching a sticker. Note that these responses are not that dissimilar from the responses used in standard perceptual tests of the SCE. Remarkably, when participants were requested to perform the same decision through object-directed movement, the SCE was eliminated. Under direct visuomotor control and grasping, numerical magnitude no longer exerted a noticeable influence on performance. The results of Experiment 2 showed that the different RT patterns under the two tasks of Experiment 1 cannot be accounted for by differences in overall response times. The results of Experiment 3 further showed that the different RT patterns under the two conditions cannot be accounted for by differences in movement trajectory. Our null effect with grasping poses a further challenge to the hypothesis of mandatory processing of numerical information.

How can one account for the collapse of the SCE in grasping? A ready explanation is based on the demonstrations that grasping is less susceptible to context than is visual perception (e.g.,^3,13,45^). Semantic (i.e., numerical magnitude) information is more easily ignored in grasping given the tight coupling of action with the dorsal stream processing^16^. Responses mediated by that system can be said to act in a more analytic fashion^13,52,53^. However, this global explanation cannot be the full story here in view of the findings that responding under visuomotor control is sensitive to numerical information (e.g.,^48,54^). For example, when people reach for objects of the *same* size, parameters of the visuomotor control are affected by numbers appear on the objects^48,55^. Therefore, the root cause of the collapse of the SCE is attentional, which already hints that the SCE is not mandatory.

The notion that numerical information is not mandatorily activated in grasping is also supported by the results of a recent study from our lab. We showed that numerical information had no effect on grasping trajectories when the target object was not visible during the reaching phase^55^. These findings support the presence of differences in processing numerical information in visual perception and visuomotor control. Future research on numeric processing should consider further dissociations between perception and action and within each system alone.

Our explanation of the present results implicates mismatched discriminability of the size and the number dimensions. This variable is of paramount importance in all selective attention and conflict tasks^14,31,56^. In the great bulk of perceptual SCE studies, the numbers were considerably more discriminable from one another than were the values of physical size^12,20,57^. Predictably enough, numerical magnitude intruded on judgements of physical size (=the SCE) more than vice versa. However, this pattern favoring number changes when the stimuli are presented as real-life 3-D objects to be grasped by the observer. In everyday life people grasp objects located in front of them. Given the real-life stimuli and the grasping task, size was likely the more salient and discriminable dimension in our study. Mismatched salience favoring size disrupted, in turn, the SCE in grasping.

Couched in the language of selectivity of attention, exclusive attention to size fails in (many) studies of visual perception because the conflicting task-irrelevant number is more salient than the task-relevant size. People are unable to ignore the salient distractor, resulting in the failure of fully selective focusing on the target size. In contrast, selective attention to size is intact in grasping real objects, because in that setup, size is more salient than task-irrelevant number. Irrelevant number is conveniently ignored, expressed as the absence of a SCE.

The current results also point to the role of the response in shaping the pattern of interaction. In particular, the activation of the response code has a retrospective effect on processing. The method by which a future response is to be conveyed affects the way a stimulus is processed. We suggest that the intention to directly interact with the target object alters the representation of the stimulus. Indeed, response mode has been shown to be one of the factors that influence relative dimensional discriminability^12,31,32^. For example, Melara and Mounts^31^ showed that the *same* words and colors (in a Stroop task) that were equally discriminable with oral responding became noticeably mismatched with manual keypress responding. Again, response mode can and does alter relative dimensional discriminability, thereby determining the outcome.

Concerning grasping, the present study highlights the role of response type in modulating basic perceptual and cognitive processing. Compared with non-directed responses, the activation of object-directed response resulted in a more analytical processing of the object’s semantic feature, making performance resistant to Stroop-like effects. Response mode can override powerful cognitive effects, casting further doubt on the automatic processing of numerical information under all circumstances.
